# Comprehensive Genome and Transcriptome Analysis Identifies SLCO3A1 Associated with Aggressive Behavior in Pigs

**DOI:** 10.3390/biom13091381

**Published:** 2023-09-12

**Authors:** Chunlei Zhang, Huan Yang, Qinglei Xu, Mingzheng Liu, Xiaohuan Chao, Jiahao Chen, Bo Zhou, Yang Liu

**Affiliations:** College of Animal Science and Technology, Nanjing Agricultural University, Nanjing 210095, China; 2020105039@stu.njau.edu.cn (C.Z.); 2021105038@stu.njau.edu.cn (H.Y.); 2019205004@njau.edu.cn (Q.X.); 2020205018@stu.njau.edu.cn (M.L.); 2021205020@stu.njau.edu.cn (X.C.); 2021105039@stu.njau.edu.cn (J.C.)

**Keywords:** CNV, aggressive behavior, *SLCO3A1*, animal welfare, dosage effect

## Abstract

Copy number variation (CNV) represents a significant reservoir of genetic diversity within the genome and exhibits a strong association with economically valuable traits in livestock. The manifestation of aggressive behavior in pigs has detrimental effects on production efficiency, immune competency, and meat quality. Nevertheless, the impact of CNV on the aggressive behavior of pigs remains elusive. In this investigation, we employed an integrated analysis of genome and transcriptome data to investigate the interplay between CNV, gene expression changes, and indicators of aggressive behavior in weaned pigs. Specifically, a subset of pigs comprising the most aggressive pigs (MAP, *n* = 12) and the least aggressive pigs (LAP, *n* = 11) was purposefully selected from a herd of 500 weaned pigs following a mixing procedure based on their composite aggressive score (CAS). Subsequently, we thoroughly analyzed copy number variation regions (CNVRs) across the entire genome using next-generation sequencing techniques, ultimately revealing the presence of 6869 CNVRs. Using genome-wide association study (GWAS) analysis and evaluating variance-stabilizing transformation (V_ST_) values, we successfully identified distinct CNVRs that distinguished the MAP and LAP counterparts. Among the prioritized CNVRs, CNVR-4962 (designated as the top-ranked *p*-value and V_ST_ value, No. 1) was located within the Solute Carrier Organic Anion Transporter Family Member 3A1 (SLCO3A1) gene. The results of our analyses indicated a significantly higher (*p* < 0.05) copy number of *SLCO3A1* in the MAP compared to the LAP. Furthermore, this increased copy number exhibited a positive correlation with the CAS of the pigs (*p* < 0.05). Furthermore, we integrated genomic data with transcriptomic data from the temporal lobe to facilitate the examination of expression quantitative trait loci (eQTL). Importantly, these observations were consistent with the mRNA expression pattern of *SLCO3A1* in the temporal lobe of both MAP and LAP (*p* < 0.05). Consequently, our findings strongly suggest that CNVs affecting *SLCO3A1* may influence gene expression through a dosage effect. These results highlight the potential of *SLCO3A1* as a candidate gene associated with aggressive traits in pig breeding programs.

## 1. Introduction

Animal welfare has emerged as a critical concern in the realm of animal production. Balancing the improvement of animal welfare with the efficiency of production remains an urgent challenge [1]. In intensive pig farms, regrouping is a commonly employed practice to optimize space utilization and enhance management efficiency [2]. However, regrouping necessitates the reestablishment of social hierarchy through aggressive encounters among pigs during the initial days of mixing [3]. This aggressive behavior inflicts severe physical harm, increases susceptibility to illness and diminishes the overall level of animal welfare [4]. It is complex for animal aggression which underlying biological regulatory mechanism. Currently, several strategies have been explored to reduce aggressive behavior in pigs, including genetic selection [5], nutritional supplementation [6], early-life socialization [7] and the use of pheromones [8]. Among these approaches, genetic selection holds the greatest potential.

Copy number variation (CNV), a prominent genetic variation class, arises from genome rearrangements, which include the deletion or duplication of DNA fragments larger than 1 Kb when compared to the reference genome [9]. Pioneering studies in the field of human genetics detected such variants within the human genome, revealing that more than 10% of individuals exhibited over 24 CNV events. These variations were believed to underlie specific phenotypic characteristics and disease susceptibilities in humans [10]. By utilizing microarray comparative genomic hybridization (aCGH), the initial map of CNVs in humans was constructed by the first study that explored CNV throughout the entire genome. This investigation identified 1447 CNV regions (CNVRs) within a cohort of 270 individuals [9].

As a valuable livestock species, pigs have also been utilized to investigate the impact of CNVRs on production traits. Previous studies on pigs’ CNV identified 37 CNVRs on chromosomes 4, 7, 14 and 17 [11]. Additionally, a comprehensive CNV survey across 18 diverse pig populations unveiled the presence of 565 CNVRs associated with various traits such as carcass length, backfat thickness (BFT), scapular length and intermuscular fat content. Notable genes linked to these CNVRs include *ANP32B*, *BSCL2*, *LTBP3* and *GDF3* [12]. Moreover, extensive investigations on CNV have been carried out in other species, including mice [13], cattle [14], sheep [15] and poultry [16].

Aggressive behavior in pigs is closely related to pig productivity and animal welfare. The primary aim of this study is to use an integrated analysis of the genome and transcriptome to identify potential CNVRs and genes involved in the regulation of aggressive behavior in pigs.

## 2. Materials and Methods

### 2.1. Sample Collection and Aggressive Behavior Observation

The experimental herd of 500 pigs utilized in this study corresponds to the same employed in our previous study [17,18]. Aggressive behavior was identified as fights or aggressive physical contact between two pigs. These aggressive behaviors are recorded as fighting if they last more than 3 s, with a duration of more than 8 s between two fights. To evaluate the aggressive behavior of each pig, we conducted observations of active attacks, which represents a fight initiated unilaterally by one pig and complied with by the other, measuring both the frequency and duration of such behavior during the initial 72 h following the mixing procedure. A composite aggressive score (CAS) was conducted, defined as the sum of the frequency of active attacks and the product of the duration of active attacks (in seconds) multiplied by 0.07. Genomic DNA was extracted from ear tissue samples using a standard phenol/chloroform/isoamyl alcohol protocol.

Based on their respective CAS values, we selected the most aggressive pigs (*n* = 12, MAP) and least aggressive pigs (*n* = 12, LAP) for next-generation sequencing (NGS) analysis from each pen; a total of 24 pigs were subjected to 150 bp paired-end read sequencing on the Illumina HiSeq2000 platform. The sequencing data underwent quality control assessment using FastQC (https://www.bioinformatics.babraham.ac.uk/projects/fastqc/, accessed on 20 December 2020), and the parameter was as follows: fastqc -o ~/out pathway/file; here, “-o” indicates the pathway of the out file, and “file” indicates the input sequencing file. Pigs with samples displaying inadequate quality were excluded from the follow-up analysis. Quality filtering and read trimming were performed using the cutadapt software (https://cutadapt.readthedocs.io/en/stable/, accessed on 30 December 2020), and the parameter was as follows: cutadapt -q 10, 15–quality-base = 33-o output file input file; here, “-q” indicates filtering the low-quality reads, “10” and “15” represent the threshold of the 3’ UTR and 5’ UTR, “–quality-base = 33” indicates the phred33 score system, “-o” indicates the pathway of the out file, and “input file” indicates the input sequencing data. MultiQC was employed to integrate the quality control results, ensuring that all samples met the requirements for subsequent CNV analysis (Figure S1A) [19]. A total of 23 samples were aligned to the Sscrofa 11.1 reference genome using the Burrows–Wheeler Aligner (BWA) (v 0.7.17) [20]. The average sequencing depth across all resequencing data was 12.92×, with a maximum of 16.07× and a minimum of 10.62×. The average alignment rate for all pigs relative to the reference genome was 98.42% (Table S1). Unless otherwise noted, the software mentioned above used default parameters for data processing.

### 2.2. CNVR Definition and Statistics

We utilized the CNVcaller software [21] to identify CNVs and CNVRs. CNVcaller utilizes Read-depth (RD) strategies to ensure high accuracy and computational efficiency. The default parameters of the software were followed for all steps of the analysis. Based on the detection results of CNVcaller, those with copy number ≤1.5 were identified as copy number loss type, those with copy number between 1.5–2.5 were identified as copy number normal type, and those with copy number ≥2.5 were identified as copy number gain type, and those with meanwhile copy number loss and copy number gain type in a DNA sequence were identified as copy number both type. Subsequently, a comprehensive CNVR map of the MAP and LAP was generated using the R package RIdeogram [22].

### 2.3. Comparison of the Detected CNVRs with Previous Studies

It is noteworthy that before 2017, CNV studies utilized the Sus Scrofa reference genome version 10.2, while the reference genome version 11.1 was adopted thereafter. Given the disparity in reference genome coordinates between the two versions, it would be inappropriate to compare studies that employed different reference genome versions. To address this concern and enable comparison of CNVR overlap rates between studies, we utilized the NCBI Genome Remapping Tool (https://www.ncbi.nlm.nih.gov/genome/tools/remap, accessed on 20 December 2020) to convert the reference genome to a uniform version (Sscrofa 11.1). By taking this approach, we were able to compare the CNVRs detected in our study with those reported in previous investigations. The tool Bedtools (v 2.15.0) was employed for the overlap analysis between different studies, following the methodology [23]. Specifically, we considered two CNVRs to overlap when they shared at least one base pair.

### 2.4. RNA Extraction and RNA-Seq

In an independent experiment, we selected the MAP (*n* = 4) and LAP (*n* = 4) from a group of 160 weaned pigs (Table S1). After dissection, the temporal lobe tissues were carefully excised and preserved in an ultra-low temperature refrigerator (Thermo Scientific, Waltham, MA, USA). Total RNA extraction was performed using the Trizol method (Vazyme, Nanjing, China). The integrity of the extracted RNA was assessed using 1% agarose gels (Vazyme, China), while RNA purity was determined via UV spectrophotometry (Thermo Scientific, USA).

### 2.5. Transcriptome Data Analysis

For the generation of sequencing libraries, we utilized the NEBNext Ultra RNA Library Prep Kit for Illumina (NEB, USA, Catalog #: E7530L), following the manufacturer’s instructions. Index codes were incorporated into the sample attribute sequences to facilitate library identification. Subsequently, eligible libraries were pooled and subjected to sequencing on the NovaSeq-6000 platform (Illumina, San Diego, CA, USA) using the PE150 strategy, guided by considerations of effective library concentration and desired data volume.

Several challenges such as splice contamination, low-quality nucleotides, and unidentifiable nucleotides (N) pose significant obstacles to ensuring reliable downstream bioinformatics analysis. To address these concerns, we employed Fastp (v 0.19.7) for quality control of raw data to obtain clean data suitable for the subsequent analysis [24]. The resulting clean data were aligned to the reference genome (Sscrofa 11.1) using HISAT2, and the featurecounts program was utilized to generate the gene expression matrix based on the clean data inputs [25,26]. Differentially expressed genes (DEGs) were identified using the R package DEseq2, employing the criteria of fold change ≥2 or ≤0.5 and a *p*-value < 0.05 [27]. Kmeans and Mfuzz methods were used to perform clustering analysis for the differential genes of the MAP and LAP [28,29]. To further explore the functional implications, gene set enrichment analysis (GSEA) and gene set variation analysis (GSVA) were conducted using the clusterProfiler and GSVA of the R package [30].

### 2.6. Gene Annotation and Enrichment Analysis

Furthermore, we utilized Chipseeker to annotate the genomic positions of the CNVRs [31]. Gene annotation and enrichment analyses were performed using g:Profiler and KOBAS, respectively [32,33].

### 2.7. CNVR Comparisons and CNV-Type Assay

A genome-wide association study (GWAS) was conducted using the mixed linear models in LDAK (v 5.2) software [34], all 6869 detected CNVRs were involved in association analysis. The mixed linear model analyzed is as follows:

y = Xa + Wb + k + e,

where y represents the phenotype vector (CAS of the MAP and LAP), a represents the vector of fixed effect (including batch and sex), b represents the allele substitution effect, k denotes the kinship matrix constructed by the LDAK-Thin algorithm, and e is the vector of random residual effects. X and W represent incidence matrices.

The degree of variation in CNVRs between the MAP and LAP was assessed using the variance-stabilizing transformation (VST) metric, which provides an unbiased measure of the fixation index (FST) [9]. The V_ST_ value was calculated using the formula:

$$V_{\mathrm{ST}}=\frac{V_{total}-\frac{V_{m}\times N_{m}+V_{l}\times N_{l}}{N_{total}}}{V_{total}},$$

In this equation, *V_total_* represents the total variance in copy number between the MAP and LAP. *V_m_* and *V_l_* correspond to the variances in copy number of the MAP and LAP, respectively. *N_m_* and *N_l_* represent the numbers of the MAP and LAP, respectively. Finally, *N_total_* denotes the total sample size. The CNVRs exhibiting the top 1% (*n* = 68) V_ST_ value were annotated using the QTL data specific to pigs, obtained from the pigs’ QTLdb (/QTLdb/SS/index).

To verify the CNV types, genomic DNA extracted from ear tissue was subjected to quantitative real-time PCR (qPCR) using the 2−ΔCT method, where ΔCt represents the difference in Ct values between the target and reference genes [35]. The highly conserved single-copy gene glucagon (GCG) in pigs was chosen as the reference gene [36]. Primers for qPCR were designed using Primer-BLAST (https://www.ncbi.nlm.nih.gov/tools/primer-blast, accessed on 20 December 2020), and the specific primer sequences are provided in Table S2. To ensure consistency between the test results of GCG and CNVR-4962, a standard curve was constructed using test samples and GCG to validate primer amplification efficiency. The qPCR experiments were performed on a QuantStudio 5 real-time PCR system (ABI, Los Angeles, CA, USA). The qPCR amplification system comprised 10 μL SYBR master mix (2×) (Vazyme, Nanjing, China), 2 μL DNA (5 ng/μL), 0.4 μL forward primer (20 pmol/μL), 0.4 μL reverse primer (20 pmol/μL) and 7.2 μL water. The qPCR program consisted of an initial step at 95 °C for 30 s, followed by 40 cycles at 95 °C for 10 s and 60 °C for 30 s. Each qPCR assay was performed in triplicate for every sample.

### 2.8. Joint Analyses of Genomics and Transcriptomics Data

The genomics and transcriptomics data joint analyses were performed via expression quantitative trait loci (eQTL) using Matrix-eQTL package, and the relationship between CNVRs and gene expression changes in the temporal lobe was evaluated [37,38].

### 2.9. Statistical Analysis

The CNVRs’ names were assigned based on their chromosome positions (Table S3). Among these, CNVR-4962 (No. 4962) was identified as a potential differential CNVR between the MAP and LAP, as indicated by its high V_ST_ value in V_ST_ analysis and higher effect value in GWAS analysis. To assess the copy number of CNVR-4962, we utilized the CNVcaller software [21] on a sample size of 23 pigs, and the results were subsequently validated through qPCR analysis involving a larger sample size of 228 pigs.

To establish the association between copy number and CAS, a two-step verification process was employed. Initially, we selected the MAP (*n* = 12) and LAP (*n* = 11) from a herd of 500 weaned pigs that had undergone next-generation sequencing (NGS). The copy numbers between these two groups were then compared using Student’s *t*-test. Subsequently, based on their copy number, the pigs were categorized into two groups: the high copy number group, comprising pigs with copy numbers in the top half, and the low copy number group, encompassing the remaining pigs. The CAS values were compared between these two groups using Student’s *t*-test. A similar grouped validation approach was applied to the qPCR-validated population, and based on their CAS values, the pigs were categorized into two groups: the MAP, comprising pigs with CAS values in the top half, and the LAP, encompassing the remaining pigs. The copy numbers between these two groups were then compared using Student’s *t*-test. In addition, we use the R package pROC for receiver operating characteristic (ROC) analysis of the possibility of CNVR-4962 as a genetic marker for pig attack behavior [39].

To analyze the CAS data, we used the GLIMMIX procedure in SAS 9.4 (SAS Institute Inc., Cary, NC, USA), with sex and batch considered as fixed effects, and pen as a random effect. This statistical analysis facilitated the examination of potential associations between CAS and copy number in a controlled manner.

## 3. Results

### 3.1. CNVR Detection and Statistics

**Figure 1:**
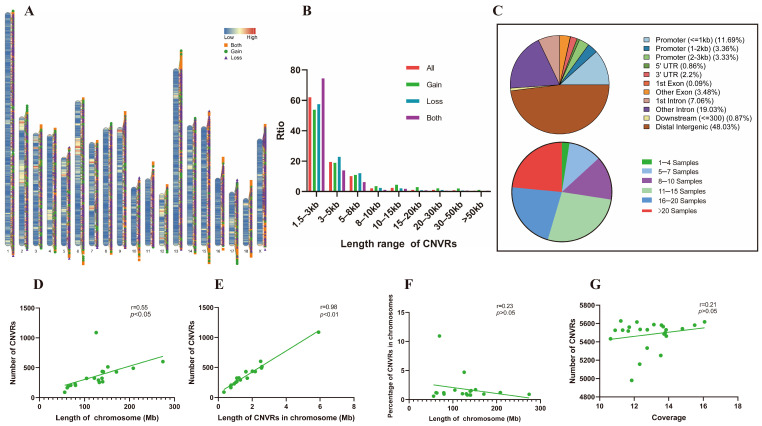
The characteristics and descriptive statistics of CNVRs. (**A**) The distribution of CNVRs and variation types of the MAP and LAP in autosomes and the X chromosome, where “Low” to “High” shows the gene density on the pig chromosomes. The yellow square, green circle and purple triangle represent the both type, gain type and loss type, respectively. (**B**) The length distribution of the CNVRs. The red, green, blue and purple colors represent all type, gain type, loss type and both type, respectively. (**C**) The distribution of positions and frequency on the genome of CNVRs. (**D**) The CNVR quantity was positively correlated with the length of the chromosomes (*p* < 0.05). (**E**) The total length of the fragment of CNVRs on each chromosome was proportional to the CNVRs quantity (*p* < 0.01). (**F**) The length of CNVRs in the chromosomes was not significantly statistically related to the length of the chromosomes (*p* > 0.05). (**G**) The sequencing depth of sequencing data was not significantly correlated with the CNVRs quantity (*p* > 0.05).

A comprehensive analysis of CNVRs in the MAP (*n* = 12) and LAP (*n* = 11) led to the detection of a total of 6869 CNVRs, comprising 3675 loss types, 2103 both types, and 1091 gain types (Figure 1A). These CNVRs collectively spanned a length of 31.07 Mb, which accounted for approximately 1.30% of the total length of chromosomes (Table S4). The length distribution of CNVRs ranged from 1.5 Kb to 194 Kb, with an average length of 4.5 Kb per section (Figure 1B). Notably, the majority of CNVRs (81.53%) were smaller than 5 Kb, while 12.32% ranged from 5 to 10 Kb, 6% fell within the 10 to 100 Kb range, and only a minor fraction (0.17%) exceeded 100 Kb in length.

Among the various types of CNVRs, those within the range of 1.5 to 3 Kb accounted for the largest proportion, with 74.42% accounting for both types, 57.47% for loss types, and 53.80% for gain types. The average lengths of both types, loss types and gain types were 3.7 Kb, 4.4 Kb and 6.4 Kb, respectively (Table S5). Genomic position annotation revealed a significant concentration of CNVRs in the “Distal Intergenic” region, followed by the “Intron”, “Promoter”, “Exon”, and “3’ and 5’ UTR” regions (Figure 1C).

Regarding the frequency distribution of CNVRs, the frequency of CNVRs appearing in 1–4 pigs was the lowest at 2.5% (169), and the frequency of CNVRs appearing in 11–15 pigs was the highest at 27.1% (1862). In total, 72.5% (4983) of CNVRs were present in more than 10 pigs (Figure 1C). These data highlight the variable occurrence and prevalence of CNVRs across the studied pig population.

### 3.2. CNVR Quantity and Chromosome Characteristics

The present study aimed to investigate the relationships between chromosome characteristics and the occurrence of CNVRs. A regression analysis revealed a significant positive association between the number of CNVRs and the length of the chromosomes (*p* < 0.05) (Figure 1D). Notably, the X chromosome exhibited a particularly high frequency of variations. Furthermore, there was a proportional relationship between the total length of CNVR fragments on each chromosome and the number of CNVRs (*p* < 0.05) (Figure 1E). However, no significant statistical correlation was observed between the length of CNVRs and the length of the chromosomes (*p* > 0.05) (Figure 1F). Additionally, the depth of sequencing data did not display a significant correlation with the number of CNVRs detected (*p* > 0.05) (Figure 1G).

### 3.3. Functional Enrichment Analysis and Comparison between the MAP and LAP

A comprehensive analysis of CNVRs was conducted in this study, with 44.6% (3067 CNVRs) demonstrating consistency with previous research findings, while the remaining 3802 CNVRs were newly identified (Table 1).

**Figure 2:**
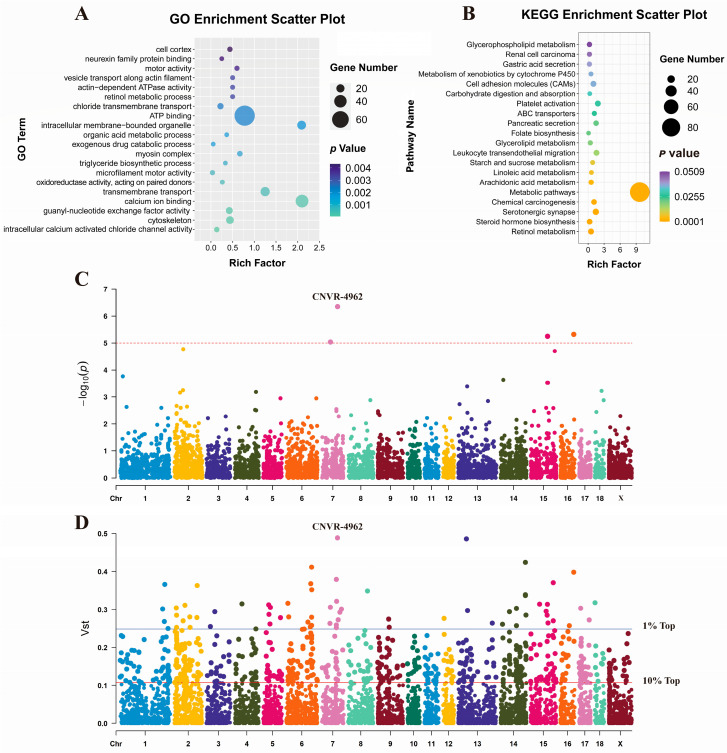
Performed enrichment analysis, GWAS analysis and V_ST_ analysis of CNVRs. (**A**,**B**) are the GO enrichment analysis and KEGG pathway analysis of all genes annotated in CNVRs, respectively. Bubbles of different colors represent different *p*-values. (**C**) GWAS analysis was used to identify CNVRs with significant effects on pig aggression. Among them, CNVR-4962 was ranked first with a *p*-value of 4.42 × 10^−7^. (**D**) Evaluate the differential CNVRs based on the V_ST_ value between the MAP and the LAP. Among them, including the CNVR-4962 (No. 1).

Subsequently, genes overlapping with CNVRs (Sscrofa 11.1) were investigated to elucidate their potential functional implications. A total of 2357 genes were found to be overlapped by the 6869 CNVRs, comprising 1188 known genes and 1169 novel genes (Table S6). To gain insights into the functional characteristics of these genes, GO and KEGG enrichment analyses were performed using the KOBAS tool. The enriched GO terms included “ATP binding”, “calcium ion binding” and “transmembrane transport”. Furthermore, the KEGG analysis revealed enrichment in pathways such as “Metabolic pathways”, “Retinol metabolism” and “Steroid hormone biosynthesis” (Figure 2A,B). Moreover, differential CNVRs in the MAP and LAP were investigated by assessing the GWAS analysis and V_ST_ value (Figure 2C,D). Notably, CNVR-4962, which overlaps with the Solute Carrier Organic Anion Transporter Family Member 3A1 (SLCO3A1) gene, exhibits significant differences between the MAP and LAP (No. 1 in the GWAS and V_ST_ analyses). *SLCO3A1* is a protein-coding gene and is thought to be associated with addictive behaviors [40].

**Table 1:** Comparison of detected CNVRs with those of previous studies based on the Sscrofa 11.1 genome assembly.

Study	Platform	Samples	Number of CNVRs (Number of CNVRs before Remapping)		Number of Overlapped CNVRs	Percent of Overlapped CNVRs from This Study (%)
[41]	Next-generation sequencing	61	12,668		1194	17.38
[42]	Next-generation sequencing	7	528 (540)		256	3.73
[43]	Next-generation sequencing	16	2614 (3118)		618	9
[44]	Next-generation sequencing	14	917 (1408)		522	7.6
[45]	Next-generation sequencing	49	2390 (3131)		142	2.07
[46]	Next-generation sequencing	240	3538		1271	18.5
[47]	1 M aCGH	12	709 (758)		829	12.07
[12]	Porcine SNP60	1693	651 (565)		648	9.43
This Study			6869			
Merge					3067	44.6

Note: CNVRs were converted to the Sscrofa11.1 version using the NCBI Genome Remapping tool. Successfully mapped CNVRs are shown in the “Number of CNVRs” column with the original number of published CNVRs (Sscrofa 10.2) shown in parentheses.

### 3.4. QTL Analysis

**Figure 3:**
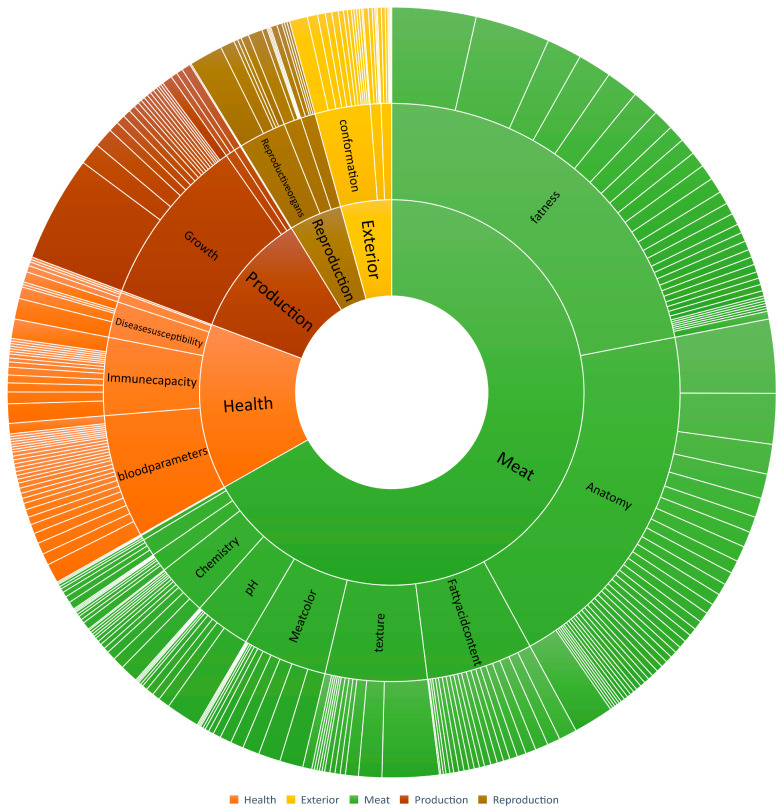
QTLs overlapped analysis of the CNVRs with the top 1% V_ST_ value.

The present study investigated the relationship between CNVRs and QTLs in pigs, with a focus on the top 1% V_ST_-ranked CNVRs (68 CNVRs) (Figure 3). Among these CNVRs, a total of 1842 QTLs were found to overlap, encompassing various traits including meat, health, production, reproduction, and exterior traits. Specifically, 1231 QTLs were associated with meat traits, 256 QTLs with health traits, 194 QTLs with production traits, 82 QTLs with reproduction traits and 79 QTLs with exterior traits.

### 3.5. Analysis of Transcriptome Data from the Temporal Lobes of the MAP and LAP

**Figure 4:**
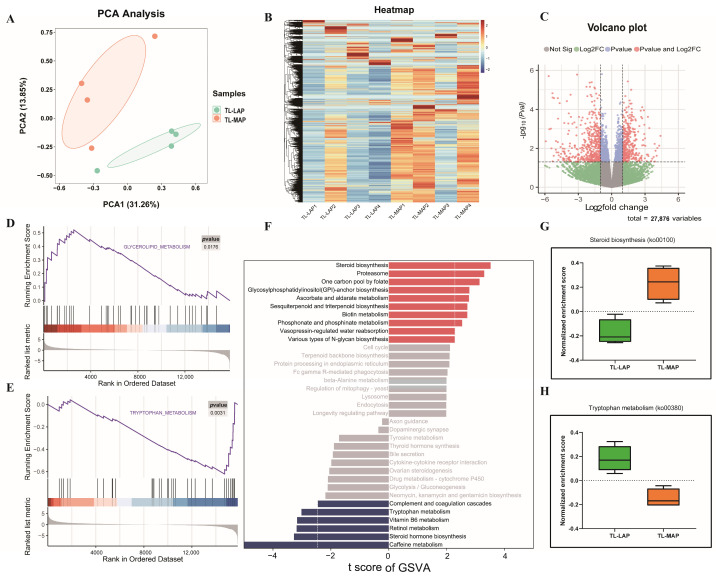
Analysis of transcriptomic data in the temporal lobes. (**A**) PCA plot of the TL-MAP and TL-LAP. TL-LAP and TL-MAP represent the temporal lobe of the LAP and the temporal lobe of the MAP, respectively. (**B**) The heat map shows the overall picture of gene expression in the TL-MAP and TL-LAP. (**C**) The volcano plot shows that there are 366 up-regulated genes and 452 down-regulated genes in the MAP relative to the LAP. (**D**,**E**) GSEA analysis showed that genes upregulated in the MAP were associated with the “Glycerolipid metabolism” pathway, while genes downregulated in the MAP were associated with the “Tryptophan metabolism” pathway. (**F**–**H**) GSVA analysis showed that the “Steroid biosynthesis” gene set was increased in the MAP, and the gene set related to “Tryptophan metabolism” upward in the LAP, TL-LAP and TL-MAP represent temporal lobe of the LAP and temporal lobe of the MAP, respectively.

Through principal component analysis (PCA) in the temporal lobe transcriptomic data of the MAP (temporal lobe of the most aggressive pigs, TL-MAP) and LAP (temporal lobe of the least aggressive pigs, TL-LAP) pigs, we found that the MAP and LAP can be clearly classified into two groups. The contribution of each PC to the genetic variation was 36.13% and 14.65% for PC1 and PC2, respectively (Figure 4A). The heat map was used to represent global gene expression changes in transcriptome data (Figure 4B). We detected 818 DEGs in the TL-MAP and TL-LAP, with 366 upregulated genes and 452 downregulated genes in the MAP compared to the LAP (Figure 4C). Based on GSEA analysis, the “Glycerolipid metabolism” pathway was closely associated with the up-regulated genes in the MAP, while the down-regulated genes in the MAP were involved in the “Tryptophan metabolism” pathway (Figure 4D,E). Based on the GSVA analysis, we found that the expression of the “Steroid biosynthesis” gene set was increased in the MAP, while the gene set related “Tryptophan metabolism” pathway was trending upward in the LAP (Figure 4F–H).

### 3.6. Cluster Analysis of DEGs in the TL-MAP and TL-LAP

**Figure 5:**
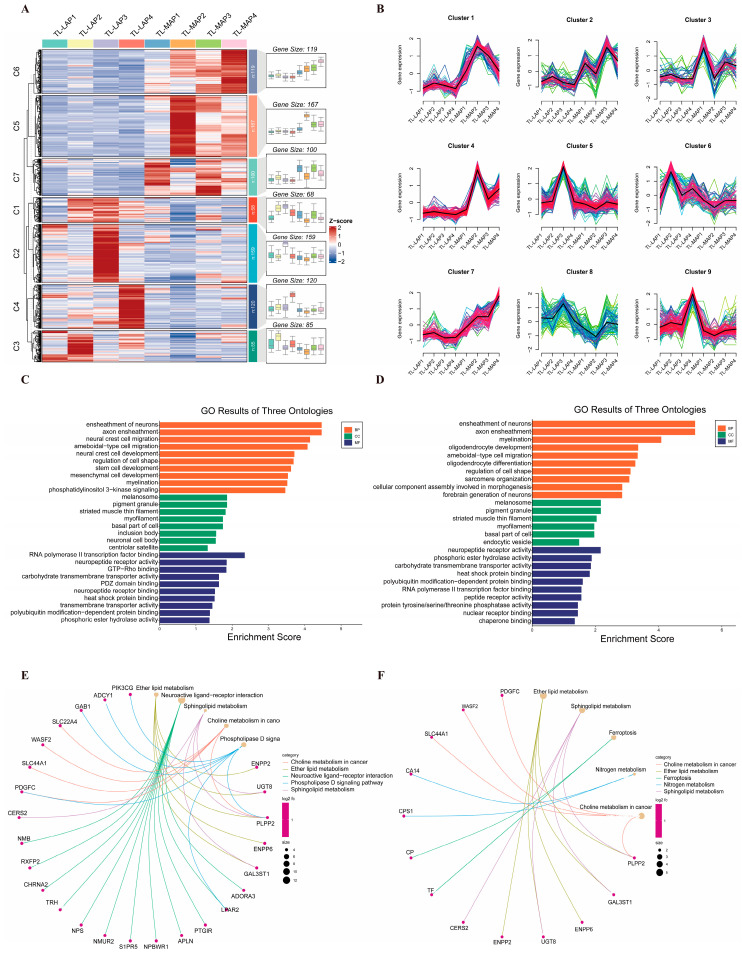
Cluster analysis of DEGs in the TL−MAP and TL−LAP. (**A**) Keans method divides the DEGs in the TL−MAP and TL−LAP into seven clusters. (**B**) The Mfuzz method divides the DEGs in the TL-MAP and TL-LAP into nine clusters. Images (**C**,**D**) represent GO enrichment analysis of genes highly expressed in the TL-MAP (clusters 5, 6 and 7 in the Kmeans method, and clusters 1, 4 and 7 in Mfuzz mothed), where “BP”, “CC” and “MF” represent the biological process, cellular component and molecular function, respectively. Images (**E**,**F**) represent KEGG pathway enrichment analysis of genes highly expressed in the TL-MAP.

A total of 818 DEGs in the TL-MAP versus the TL-LAP were categorized into seven clusters and nine clusters via Keans and Mfuzz cluster analysis (Figure 5A,B). In Keans cluster analysis, clusters 5, 6 and 7 contained 167, 119 and 100 genes, respectively. These genes are up-regulated in expression in the TL-MAP (Figure 5A). GO enrichment analysis of these genes revealed that they are related to “ensheathment of neurons”, “neuronal cell body”, “neuropeptide receptor activity” in the biological process, cellular component and molecular function (Figure 5C). But in clusters 1, 2, 3 and 4, these terms are associated with “thyroid hormone metabolic process”, “apical part of cell” and “exopeptidase activity” in the biological process, cellular component and molecular function, respectively (Figure S1B). In Mfuzz cluster analysis, A total of 276 genes in cluster 1, 4, 7 were highly expressed in the TL-MAP (Figure 5B). These genes were associated with neuronal cell development and lipid metabolism in GO and KEGG pathway enrichment analyses (Figure 5D,F).

In addition, genes in cluster 5, 6 and 7 were shown to be associated with “Neuroactive ligand-receptor interaction” in the KEGG pathway enrichment analysis (Figure 5E), while genes in clusters 1, 2, 3 and 4 were analyzed as being associated with “Cytokine-cytokine receptor interaction” (Figure S1C). Cluster 8 contained 85 genes that were highly expressed in the LAP. GO and KEGG enrichment analyses indicated that these genes were associated with the Cytoskeleton and protein absorption (Figure S1D,E).

### 3.7. Multi-Omics Analysis Reveals a Potential Relationship between the SLCO3A1 Gene and Aggressive Behavior in Pigs

**Figure 6:**
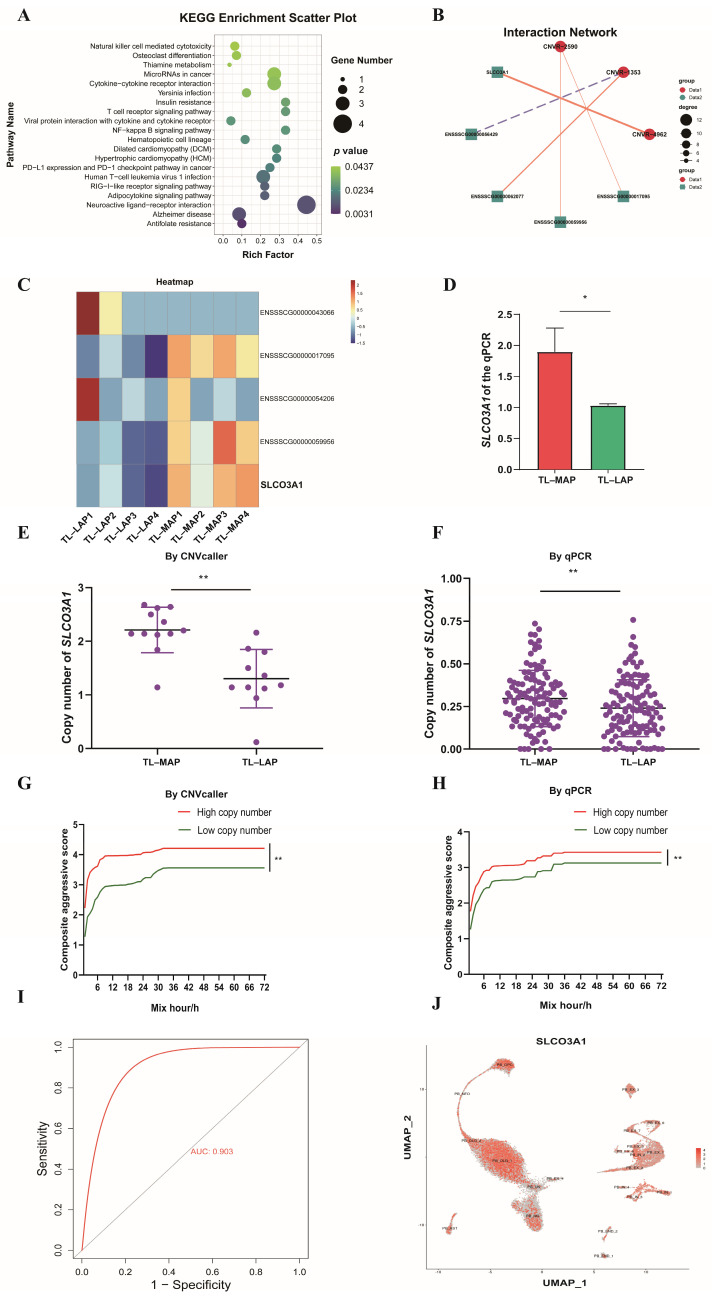
Integrated analysis of the genomic and transcriptomic data. (**A**) 407 trans-eQTL genes are associated with the “Alzheimer disease” and “Neuroactive ligand-receptor interaction”. (**B**) Interaction network of 3 CNVRs and 5 neighboring genes with eQTL effects; the yellow line indicates positive regulation, and the purple line indicates negative regulation. Graphs (**C**,**D**) represent the relative expression in the *SLCO3A1* in the TL-MAP and TL-LAP, and (**C**,**D**) are based on transcriptomic data and qPCR mothed, respectively. Images (**E**,**F**) are the evaluation results of the copy number of *SLCO3A1* via CNVcaller and qPCR (via qPCR: *n* = 228). Images (**G**,**H**) are the relationship between the copy number of *SLCO3A1* and CAS. (**I**) ROC curves of *SLCO3A1* copy number as a genetic marker in the MAP and LAP. (**J**) Single-cell transcriptome data showed extensive expression of *SLCO3A1* in the frontal, parietal, temporal and parietal lobes, and in the hypothalamus. * represents *p* < 0.05, ** represents *p* < 0.01.

To investigate the potential impact of differential CNVRs on gene expression changes, the top 10 V_ST_-ranked CNVRs (Table S7) were selected for eQTL analysis with all genes detected in the transcriptome data. A total of 407 genes were identified as potential candidates for expression changes mediated by trans-eQTL effects of CNVRs (Table S8). These genes were found to be involved in pathways such as “Alzheimer disease” and “Neuroactive ligand-receptor interaction” (Figure 6A). Additionally, a cis-eQTL analysis revealed putative interactions between 3 CNVRs and 5 neighboring genes, indicating their potential regulatory relationships (Figure 6B). These findings provide insights into the intricate interplay between CNVRs and gene expression changes in the context of aggressiveness traits in pigs.

The porcine *SLCO3A1* gene is located at chr7: 86,777,919 to 87,103,316 (Sscrofa 11.1); it overlaps with CNVR-4962: chr7: 86,862,001 to 86,864. The qPCR methods revealed increased *SLCO3A1* mRNA in the aggressive group, which concurred with the expression pattern of *SLCO3A1* mRNA observed in the transcriptome data (higher expression in the TL-MAP compared to the TL-LAP) (Figure 6C,D).

To assess the relationship between the CAS and the copy number of *SLCO3A1*, the copy number of *SLCO3A1* was evaluated via CNVcaller and verified through qPCR in an additional 228 pigs. Based on CNVcaller and qPCR evaluations, there was a significantly higher copy number of *SLCO3A1* in the MAP compared to the LAP (Figure 6E,F). Furthermore, the copy number of *SLCO3A1* exhibited a positive correlation with the CAS of pigs after mixing (Figure 6G,H). The copy number of *SLCO3A1* detected by CNVcaller was subjected to ROC analysis, and its area under the roc curve (AUC) was 0.903, indicating that the copy number of *SLCO3A1* can be a putative molecular marker for determining the aggressiveness of pigs (Figure 6I). Moreover, a single-cell sequencing data from the frontal cortex, temporal cortex, parietal cortex, occipital cortex, and hypothalamus of pigs was reanalyzed and found widespread distribution of *SLCO3A1* in the porcine brain [48] (Figure 6J), which suggests that *SLCO3A1* may play an important role in the regulation of aggressive behavior in pigs.

## 4. Discussion

In this study, we detected a total of 6869 CNVRs in 23 wean pigs exhibiting aggressive behavior, representing approximately 1.30% of the porcine reference genome (Sscrofa 11.1). The CNVRs associated with aggressive behavior in pigs were analyzed using extensive genome-wide sequencing data.

Among the CNVRs identified, the frequency of loss-type CNVRs (3675) was higher than that of gain-type CNVRs (1091) in pigs. This finding aligns with a previous study that reported a higher frequency of loss-type CNVRs (243) compared to gain-type CNVRs (88) in Chinese and European pigs [49]. However, a different pattern was observed in Chinese Xiang and Kele Pigs, where the frequency of gain-type CNVRs (97) exceeded that of loss-type CNVRs (65) [50]. Another CNV study also reported similar results with 4558 gain-type CNVRs and 3724 loss-type CNVRs [41]. To compare the overlapping CNVRs with previous studies, we converted the pig’s reference genome to the same version (Sscrofa 11.2). The overlapped CNVRs ranged from 2.07% (142) to 17.38% (1194) in different studies [40,47], indicating substantial variation among studies. This suggests that the repeatability and accuracy of different CNV studies are influenced by factors such as pig breeds, CNV detection methods, or software used. Although this remapping method is also influenced by the factors mentioned above, leading to inaccurate results, it can still provide information for comparisons between different studies. The task of detecting CNVs in the genome with increased simplicity, cost effectiveness, and precision continues to pose an unresolved query in the field.

This study also investigated the relationship between CNVRs and chromosome characteristics. Our findings revealed a positive association between the number of CNVRs and chromosomal length, consistent with previous studies [46,51]. We identified a CNV hotspot region on the X chromosome in our study, whereas previous studies reported CNV hotspot regions on chromosomes 13 and 6 [45,52].

The temporal lobe, known for its involvement in memory, emotion, social interaction and emotional communication [53], has been implicated in aggression. Experimental studies in rats have demonstrated an increase in aggression when controlling for temporal lobe lesions [54]. Similarly, in humans, the removal of temporal lobe tumors resulted in a significant improvement in aggressive behavior [55]. In our study, we observed significant gene expression changes in the TL-MAP and TL-LAP, suggesting its potential role in regulating aggressive behavior. Further investigation of these genes exhibiting altered expression in the temporal lobe will enhance our understanding of the underlying mechanisms governing aggressive behavior.

Through GSEA and GSVA analyses, we identified highly expressed genes associated with the glycerol metabolic and steroid biosynthesis pathway in the MAP. Glycerol metabolites and steroidal substances, such as glucocorticoids and unsaturated fatty acids, are strongly associated with aggressive behavior [56,57]. Conversely, we observed high expression of genes related to the tryptophan metabolic pathway in the LAP. Tryptophan, widely recognized for its sedative and relaxing properties, has been shown to reduce aggressive behavior in fish and mice upon exogenous administration [58,59]. Serotonin, a metabolite of tryptophan, belongs to a class of neurotransmitters that can mitigate aggressive behavior. The modulation of serotonin levels or serotonin transporters has been found to regulate aggressive behavior [60,61].

In our present study, we examined the differential CNVRs between the MAP and LAP using the GWAS and V_ST_ analysis. Among these CNVRs, we discovered a significant difference in CNVR-4962 (By *p*-value and V_ST_ value), which overlaps with the *SLCO3A1* gene. This finding aligns with the expression pattern of *SLCO3A1* in the temporal lobe, indicating that CNVR-4962 may influence *SLCO3A1* expression through dosage effects, potentially contributing to aggressive behavior in pigs.

The Solute Carrier Organic Anion Transporter (SLCO) family encompasses transporters that facilitate the entry of various compounds into cells. Their expression in diverse tissues such as the bowel, liver, kidney and brain suggests their pivotal role in metabolism, immunity and neural development [62]. Previous investigations have focused on understanding the recognition and functional characteristics of SLCOs. For instance, functional SNPs of *SLCO1B1* have been shown to decrease hepatic uptake activity [63], while the expression level of *SLCO2B1* substantially declined in the small intestine of rats with acute kidney injury [64]. The protein product of *SLCO3A1* was found to be enriched in glial cells and neurons, particularly in the gray matter axons of frontal cortical neurons [65], suggesting a potential association with neuronal signal conduction re-uptake mechanisms. In humans, *SLCO3A1* variants have been implicated in regulating extracellular vasopressin concentration, potentially influencing neurotransmission mediated by cerebral neuropeptides such as vasopressin [66]. The deletion of *SLCO3A1* fragments has been strongly linked to Angelman syndrome (AS), a severe cognitive disorder characterized by ataxia, epilepsy and abnormal behavior [67]. Additionally, a genome-wide association study identified *SLCO3A1*’s involvement in nicotine dependence, highlighting its relevance to addiction [68].

Considering the pivotal role of *SLCO3A1* in neurodevelopment and mental illness, we hypothesize that it holds regulatory potential in the context of aggressive behavior in pigs. The assessment of gene CNV and its effects is often conducted using the qPCR method. Noteworthy examples include the association of AHR gene CNV with reproductive traits in Meishan pigs [41], the correlation of MTHFSD gene CNV with litter size traits across pig breeds [36] and the involvement of MSRB3 gene CNV in porcine ear size [69]. In line with this, our qPCR analysis of *SLCO3A1* revealed a significant association between its CNV and aggressive behavior in weaned pigs following mixing. Specifically, the copy number of *SLCO3A1* was markedly higher in the MAP pigs compared to the LAP ones. Our comprehensive CNV analysis expands the CNV landscape of the pig genome, providing valuable insights into the genomic structure of genetic variations underlying pig aggressive behavior. These findings have the potential to reduce pig aggression and enhance animal welfare in marker-assisted genomic selection programs.

## 5. Conclusions

In conclusion, our study employed an integrated analysis of genome and transcriptome data to investigate the interplay between CNV, gene expression changes, and indicators of aggressive behavior in weaned pigs. Through the evaluation of GWAS analysis, V_ST_ values, and relative copy numbers, we have identified *SLCO3A1* as a potential CNV-gene candidate associated with aggressive behavior in pigs. The CNV of *SLCO3A1* likely exerts regulatory control over its gene expression in the temporal lobe through dosage effects. These results contribute to our understanding of the underlying regulatory mechanisms through which CNVs influence aggression in pigs.

## Data Availability

The datasets presented in this study can be found in online repositories. The names of the repository/repositories and accession number(s) can be found in the article/Supplementary Materials.

## Supplementary Materials

The following supporting information can be downloaded at https://www.mdpi.com/article/10.3390/biom13091381/s1. Supplementary Table S1: The whole-genome resequencing data of the MAP and LAP. Supplementary Table S2: The standard curve and primers of qPCR. Supplementary Table S3: A total of CNVRs and CNV types. Supplementary Table S4: Distribution of CNVRs on chromosomes of the MAP and LAP. Supplementary Table S5: The length distribution and proportion of the CNVRs. Supplementary Table S6: Annotated genes in CNVRs of MAP and LAP. Supplementary Table S7: CNVRs information with top 10 V_ST_ value. Supplementary Table S8: Genes with trans-eQTL effect. Supplementary Figure S1: Genomic data quality and GO and KEGG enrichment analysis of DEGs between the TL-MAP and TL-LAP.
